# Monitoring game load across quarters in professional basketball: positional differences and contextual effects

**DOI:** 10.5114/biolsport.2026.157997

**Published:** 2026-01-23

**Authors:** Shaoliang Zhang, Ming Li, Xing Wang, Enrique Alonso-Pérez-Chao, Pierpaolo Sansone, Jordan L Fox

**Affiliations:** 1 Athletic Performance and Data Science Laboratory (APDS), Division of Sports Science and Physical Education, Tsinghua University, Beijing, China; 2 School of Athletic Performance, Shanghai University of Sport, Shanghai, China; 3 Department of Physical Activity and Sports Science, University Alfonso X el Sabio, Villanueva de la Cañada, Spain; 4 Department of Movement, Human and Health Sciences, University of Rome “Foro Italico”, Rome, Italy; 5 Allied Health and Human Performance, University of South Australia, Adelaide, Australia

**Keywords:** Basketball, External and internal loads, Game quarters, Playing position, Performance, Contextual factors

## Abstract

This study aimed to examine quarter-to-quarter variations in external and internal loads, the relationships between RPE and external loads, and the effects of contextual factors on external and internal loads in backcourt and frontcourt players. 16 professional male basketball players (8 backcourt, 8 frontcourt) from the Chinese National Basketball League were recruited. External load was quantified using Catapult S7 devices to record PlayerLoad^TM^ (PL) and Inertial Movement Analysis (IMA) metrics, while internal load was assessed via rating of perceived exertion (RPE). From the first to the fourth quarter, frontcourt players displayed significant reductions in change of direction to the right (Q1: 0.48 ± 0.29 vs. Q4: 0.33 ± 0.16; p < 0.05) and explosive efforts (Q1: 1.49 ± 0.40 vs. Q4: 1.04 ± 0.35; p < 0.01). In contrast, backcourt players reported higher RPE (Q1: 4.28 ± 1.04 vs. Q4: 5.13 ± 1.05; p < 0.05) and recorded higher PL (Q1: 103.10 ± 44.59 vs. Q4: 123.21 ± 57.66; p < 0.01) in the fourth quarter. Frontcourt players showed higher RPE, PL, and PL · min^−1^, especially in the second quarter, but lower jump-related (IMA jump low, medium, total) and acceleration metrics than backcourt players. RPE correlated moderately to largely with PL and high-intensity jumps (r = 0.41–0.72, all p < 0.05). Backcourt players reported higher RPE in wins compared with losses (ES = 0.42; p < 0.05). These findings reveal notable positional differences in game load profiles, underscoring the need for contextspecific load management to optimize position-specific recovery.

## INTRODUCTION

Physical performance in basketball games is shaped by multiple factors, particularly technical-tactical and contextual influences. [1]. As the game pace and intensity fluctuate, players face not only higher physical demands but also more complex environmental challenges (e.g., score margin or game location) across game quarters [2]. Furthermore, playing positions, such as backcourt and frontcourt, are linked to distinct load patterns [3]. Therefore, understanding how positional demands vary across game quarters is essential for optimizing training and competition, with direct implications for coaching practice and performance management.

Previous research has consistently shown that game-related load volume and intensity vary by playing position and across game quarters [3,4,5]. Backcourt players complete more frequent decelerations (DEC) compared to frontcourt players in professional male basketball [4] whereas frontcourt players achieve higher session ratings of perceived exertion (sRPE) compared to backcourt players in youth male basketball [5]. Beyond positional differences, basketball players also experience fluctuations in load across game quarters. For example, previous studies have identified decreases in the high-intensity efforts from the first to the fourth quarters in professional male basketball players [6]. Furthermore, a previous study analyzed average external demands in official U18 basketball games with overtime, reporting significant increases in total distance and PlayerLoad™ (PL) during overtime compared with the final quarter [7]. Similarly, prior studies involving junior and professional male basketball players have reported significant declines in peak demands across games, with the largest reductions in total distance, PL, and high-speed running observed between the first and fourth quarters [8, 9]. A recent study extended this analysis to elite male basketball players competing in the Eurocup, observing a small to moderate decline in peak intensities for PL across game quarters [10]. However, how load varies across game quarters in relation to playing position remains unknown. While some studies examine player positions, they often overlook temporal dynamics across game quarters [10–12]. Conversely, research focusing on inter-period variations frequently fails to distinguish these changes by specific player positions [7, 8, 13]. Therefore, further investigation addressing this gap is warranted.

Understanding how game load varies by playing position across game quarters is critical for developing effective training and game strategies. More specifically, establishing the dose–response relationships between external and internal loads across game quarters is essential for optimizing player performance and adaptations [14]. In basketball, research has shown significant, large correlations between sRPE and PL (r = 0.53) and moderate associations between sRPE and acceleration (ACC, r = 0.40), DEC (r = 0.39), and change of direction (COD, r = 0.35) counts during gameplay in semiprofessional male basketball players [14]. Importantly, the relationship between sRPE and external load appears to vary by position [5, 15]. Specifically, youth male backcourt players exhibit small correlations (r = 0.18) between sRPE and Impulse Load (representing the total area under 3-axis accelerometer curves, accounting for locomotor events and impacts), while youth male frontcourt players show moderate correlations (r = 0.30) [5]. However, no study has previously examined positional changes in these external-internal load relationships across game quarters. This gap limits a comprehensive understanding of how external and internal loads interact across a game, highlighting the need for further investigation.

Contextual factors, also known as situational variables, strongly influence game performance in basketball [16]. Factors such as game location, scoring line, and opponent level a significant influence on game-related loads in basketball [17–19]. Playing against higher-ranking opponents may reduce PL·min^-1^ and total PL, while sRPE tends to be higher in away than in home games in semi-professional male basketball players. [16, 20]. However, how contextual factors differentially affect physical demands of backcourt and frontcourt players remains unexplored. Given the importance of player rotation and load distribution for performance management, it is essential to understand how positional characteristics interact with contextual factors across the course of a game [21].

Given these considerations, the current study aims to: 1) examine the variations in external and internal loads between- and within-quarters for backcourt and frontcourt players; 2) explore the relationships between RPE and external loads across quarters for frontcourt and backcourt players; and 3) explore the influence of contextual factors (e.g. game location, the scoring line, and opponent level) on external and internal loads in backcourt and frontcourt players. We hypothesize that backcourt players may exhibit higher PL and RPE during early game quarters compared to their frontcourt counterparts. Furthermore, the association between RPE and PL increased across game quarters.

## MATERIALS AND METHODS

### Participants

A total of 16 professional male basketball players [backcourt (mean ± standard deviation (SD): age: 28.4 ± 1.2 years; height: 197.3 ± 2.4 cm; body mass: 101.4 ± 8.7 kg, N = 8); frontcourt (age: 26.2 ± 1.7 years; height: 209.6 ± 2.3 cm; body mass: 118.5 ± 3.7 kg, N = 8) participated in this study. All players were from the same team competing in the Chinese National Basketball League, a second-tier, professional level competition.

To ensure data reliability and representativeness, only players with an average playing time of at least 15 minutes per game and a minimum of 5 minutes per quarter were included in the analysis based on previous studies [22]. Quarter-level data were retained only if these criteria were met in at least five games. Participants underwent comprehensive health screenings to confirm the absence of musculoskeletal, neurological, or orthopedic injuries, ensuring compliance with experimental standards. All players demonstrated proficiency in using digital questionnaires and wearable devices, and their participation was closely monitored in alignment with the coaching staff to ensure consistent game involvement. Written informed consent was obtained from all participants, who were fully informed on their rights and responsibilities prior to the study. The study adhered to the ethical principles outlined in the Declaration of Helsinki and received approval from the Institutional Review Board of Tsinghua University, Beijing, China.

### Study design

This longitudinal observational study was conducted across games 2 to 20 of the 25-game regular season. Each game consisted of four 10-minute quarters, with playing time measured inclusive of all active stoppages, specifically fouls and out-of-bounds events, but excluded time-outs, substitutions, and inter-quarter breaks. Moreover, a posteriori (post hoc) power analysis conducted using G*Power (version 3.1.9.6, Germany) showed that at least 14 participants were required to detect a medium effect size (ES = 0.25) with a power of 0.80 and α = 0.05 in repeated-measures analyses based on prior research [23], confirming that the current sample size was statistically adequate. A total of 520 individual player observations were collected and distributed as follows: 120 in the first quarter (Q1), 150 in the second quarter (Q2), 148 in the third quarter (Q3), and 102 in the fourth quarter (Q4).

### External and Internal Load Measurement

Each player wore a Catapult S7 device (Catapult Innovations; Melbourne, Australia) to monitor external load during games. These devices are equipped with a 100-Hz tri-axial accelerometer, gyroscope, and magnetometer and were positioned between the scapulae at the level of the C7–T1 vertebrae using a manufacturer’s vest [24]. PL (arbitrary units, AU), a key external load variable derived from accelerometer data was calculated both as an absolute value and relative to playing time (PL · min^−1^). After each game, data were downloaded and exported at 100 Hz and reported as accumulated PL, representing the square root of the change in acceleration across the transverse (x), coronal (y), and sagittal (z) planes, calculated using proprietary software (OpenField version 3.10.5; Catapult Innovations) via the following formula: PL = [√ (Ac1n – Ac1n-1)^2^ + (Ac2n – Ac2n-1)^2^ + (Ac3n – Ac3n1)^2^] × 0.01, where Ac1, Ac2, and Ac3 are the orthogonal components measured by the tri-axial accelerometer, and 0.01 is the scaling factor [25]. The reliability of PL has been previously provided in team sport contexts with coefficient of variation (CV) ranging between 0.9–1.9% [26].

To quantify external load in detail, inertial movement analysis (IMA) variables were also captured from the wearable devices and categorized by directional player movements and intensity levels. Directional movements included ACC (-45° to 45°), DEC (−135° to 135°), COD to the left (-135° to -45°), and COD to the right (45° to 135°). These movements were further classified by intensity as low (1.5–2.5 m · s^−2^), medium (2.5–3.5 m · s^−2^), and high (> 3.5 m · s^−2^) according to proprietary thresholds previously used in basketball research [14]. Vertical jumps, detected using proprietary algorithms, were categorized by height into low (< 20 cm), medium (20–40 cm), and high (> 40 cm) intensity events [27]. Explosive efforts were defined as high-intensity IMA events (> 3.5 m · s^−2^) and included the sum of IMA ACC, DEC, and COD events. Thresholds for IMA variables were selected based on proprietary methods in basketball research, as highlighted in a recent review [28]; however, low and medium-intensity IMA ACC, DEC, and COD metrics were excluded from our analyses due to significant multicollinearity (p < 0.001) with at least one high-intensity IMA-related variable, in order to avoid inflating the number of variables in the statistical models [29–32].The reliability of IMA-derived external load measures in team sports has been previously validated, with coefficients of variation ranging from 3.1% to 6.7% [33].

To assess internal load, players reported individualized RPE using the modified CR-10 scale after each quarter [34]. In the current present analysis, RPE was employed instead of sRPE-load to minimize the potential confounding effects introduced by playing time [1]. This method, widely utilized across team sports, has been shown to strongly correlate with objective physiological measures such as blood lactate concentration and heart rate [35]. RPE data was collected via an online survey platform (Google Forms, CA, USA).

### Contextual factors

Opponent level was determined based on the team’s winning percentage [19], using k-means cluster analysis to classify teams into two categories: weak teams (42.5 ± 6.8%) and strong teams (63.5 ± 8.4%).

Similarly, k-means clustering was applied to classify games by final score differences into two groups: balanced (1–16 points) and unbalanced (> 16 points) games [17]. Game outcomes were categorized as either a win or a loss [16].

### Statistical Analysis

All data are presented as mean ± SD. All analyses were conducted in R Studio (version 3.5.3; R Foundation for Statistical Computing, Vienna, Austria). To examine between- and within-quarter variations according to player position during competition, linear mixed-effects models were fitted using the R packages *lme4, lmerTest*, and *report*, with “quarter” (Q1–Q4) and “position” (backcourt, frontcourt) specified as fixed effects, and “Player ID” and “Game code” included as random effects [36]. Model assumptions were carefully evaluated: normality of residuals via Q–Q plots, homoscedasticity via residualversus-fitted plots, and independence through the random-effects structure and inspection of residual autocorrelation [37]. These diagnostics confirmed that all assumptions were adequately satisfied, supporting the validity of the mixed-effects analyses.

To account for the non-independence of repeated observations within players and to evaluate within-player associations between RPE and external load metrics across quarters, repeated-measures correlation analyses were conducted using the R package *rmcorr* [14]. This method estimates a common within-individual correlation while controlling for between-subject differences in means, making it appropriate for datasets with multiple measurements per participant [38]. Analyses were performed separately by playing position (backcourt and frontcourt) to examine the relationships between RPE and external load variables. For each position, correlation coefficients with 90% confidence intervals and p-values were reported. Correlation measures were interpreted using the following criteria: trivial: < 0.10; small: 0.10–0.29; medium: 0.30–0.49; large: 0.50–0.69; very large: 0.70–0.89; nearly perfect: 0.90–1.00 [39].

Finally, to extend these analyses and evaluate the influence of contextual factors (e.g., scoring line, opponent level, and game outcome) on backcourt and frontcourt players, generalized estimating equations (GEE) were employed in addition to mixed-effects models. Whereas mixed-effects models provide subject-specific estimates, GEE yields population-averaged effects and is more robust to deviations from distributional assumptions [40]. GEE models were fitted for repeated measures to estimate the effects of contextual variables on continuous load metrics. Marginal means for each categorical factor (e.g., balanced vs. unbalanced scoring line, strong- vs. weak opponent, and win vs. loss) were obtained using the R package *emmeans*. Pairwise contrasts between levels were extracted and standardized into Cohen’s d, with Hedges’ g correction applied for small-sample bias. Confidence intervals (90% CI) were derived from the standard error of the contrasts, and forest plots were used to present effect sizes and their confidence intervals across all load metrics under contextual factors. Effect sizes (ES) were computed and interpreted as follows: trivial: 0–0.19; small: 0.2–0.59; moderate: 0.6–1.19; large: 1.2–1.99; > 2.0 very large. Statistical significance was set at p < 0.05 [39].

## RESULTS

**FIG. 1:**
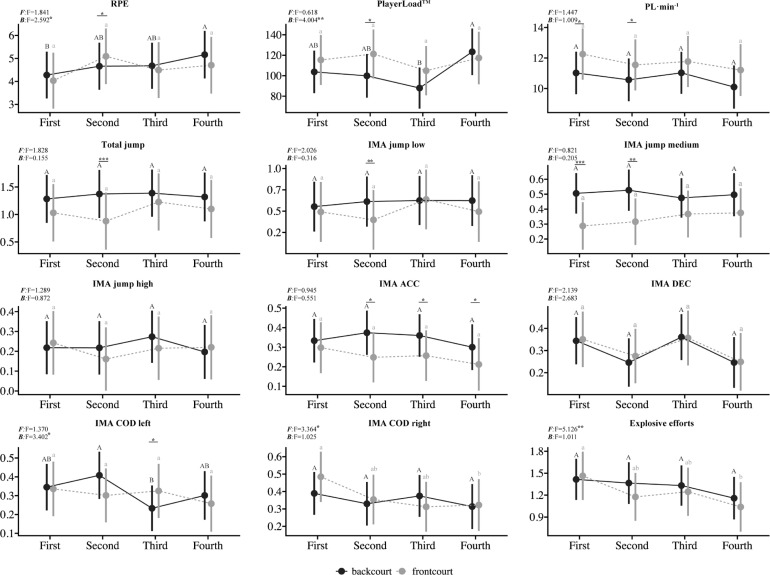
The comparison of external and internal loads between and within quarters based on backcourt and frontcourt players. Note: Different letters indicate significant differences between quarters within the same positional group. Asterisks indicate significant differences between backcourt and frontcourt players within the same quarter; RPE, rating of perceived exertion; PL, PlayerLoad; ACC, acceleration; DEC, deceleration; COD, change of direction; *p ≤ 0.05, **p ≤ 0.01, ***p ≤ 0.001

Table 1 and Figure 1 summarize external and internal loads across game quarters for backcourt and frontcourt players.

**TABLE 1:** Descriptive statistics of external and internal loads across game quarters based on backcourt and frontcourt players

Variables	First quarter		Second quarter		Third quarter		Fourth quarter	

	Backcourt	Frontcourt	Backcourt	Frontcourt	Backcourt	Frontcourt	Backcourt	Frontcourt
RPE, AU	4.28 ± 1.04	4.16 ± 1.17	4.57 ± 1.02	5.11 ± 1.27	4.62 ± 1.21	4.47 ± 1.20	5.13 ± 1.05	4.52 ± 1.29

PlayerLoad, AU	103.10 ± 44.59	116.06 ± 47.80	99.85 ± 36.37	121.41 ± 46.39	87.93 ± 34.35	105.50 ± 39.06	123.21 ± 57.66	117.84 ± 53.31

PL · min−1, AU · min−1	11.08 ± 1.82	12.11 ± 2.06	10.32 ± 1.61	11.43 ± 1.64	10.82 ± 1.95	11.69 ± 1.84	10.10 ± 1.94	11.27 ± 2.56

Total jump, count · min−1	1.28 ± 0.60	1.04 ± 0.40	1.32 ± 0.63	0.89 ± 0.35	1.36 ± 0.64	1.25 ± 0.66	1.31 ± 0.83	1.06 ± 0.85

IMA jump low, count · min^−1^	0.57 ± 0.35	0.48 ± 0.26	0.60 ± 0.42	0.39 ± 0.22	0.63 ± 0.45	0.64 ± 0.54	0.67 ± 0.56	0.49 ± 0.48

IMA jump medium, count · min^−1^	0.50 ± 0.31	0.28 ± 0.18	0.51 ± 0.30	0.31 ± 0.21	0.46 ± 0.27	0.36 ± 0.23	0.50 ± 0.29	0.37 ± 0.29

IMA jump high, count · min^−1^	0.21 ± 0.17	0.28 ± 0.18	0.21 ± 0.17	0.16 ± 0.10	0.27 ± 0.23	0.21 ± 0.19	0.20 ± 0.18	0.22 ± 0.20

IMA ACC, count · min^−1^	0.33 ± 0.22	0.29 ± 0.16	0.37 ± 0.24	0.25 ± 0.16	0.36 ± 0.22	0.26 ± 0.24	0.32 ± 0.19	0.21 ± 0.17

IMA DEC, count · min^−1^	0.34 ± 0.23	0.36 ± 0.19	0.24 ± 0.12	0.28 ± 0.20	0.37 ± 0.32	0.36 ± 0.20	0.25 ± 0.16	0.26 ± 0.18

IMA COD left, count · min^−1^	0.35 ± 0.23	0.34 ± 0.22	0.40 ± 0.32	0.29 ± 0.15	0.23 ± 0.18	0.33 ± 0.18	0.31 ± 0.24	0.25 ± 0.16

IMA COD right, count · min^−1^	0.39 ± 0.24	0.48 ± 0.29	0.32 ± 0.22	0.37 ± 0.15	0.38 ± 0.24	0.32 ± 0.28	0.31 ± 0.16	0.33 ± 0.16

Explosive efforts, count · min^−1^	1.40 ± 0.57	1.49 ± 0.40	1.33 ± 0.53	1.19 ± 0.41	1.32 ± 0.53	1.27 ± 0.52	1.19 ± 0.42	1.04 ± 0.35

Note: RPE, rating of perceived exertion; PL, PlayerLoad; ACC, acceleration; DEC, deceleration; COD, change of direction; AU, arbitrary units.

Frontcourt players demonstrated a significant decline in IMA COD right (F = 3.364, p < 0.05) and explosive efforts (F = 5.126, p < 0.01) from the first quarter to the fourth quarter. Conversely, backcourt players experienced a substantial increase in RPE during the fourth quarter relative to the first quarter (F = 2.592, p < 0.05). PL for backcourt players also increased significantly in the fourth quarter compared to the third quarter (F = 4.004, p < 0.01) but IMA COD left decreased during the third quarter compared with the second quarter (F = 3.402, p < 0.05).

Within-quarter analyses (Figure 1) revealed significant positional differences. In the first quarter, frontcourt players exhibited significantly higher PL · min^−1^ (p < 0.05) but lower IMA jump medium (p < 0.001) compared to backcourt players. In the second quarter, frontcourt players demonstrated significantly higher RPE values (p < 0.05), PL (p < 0.05), and PL · min^−1^ (p < 0.05) but significantly lower total jump (p < 0.001), IMA jump low (p < 0.05), IMA jump medium (p < 0.05), and IMA ACC (p < 0.05) compared to their backcourt counterparts. During the third quarter, frontcourt players showed significantly lower IMA ACC (p < 0.05) but higher IMA COD left (p < 0.01). In the fourth quarter, frontcourt players exhibited a significant reduction in IMA ACC (p < 0.05) compared to backcourt players.

**FIG. 2:**
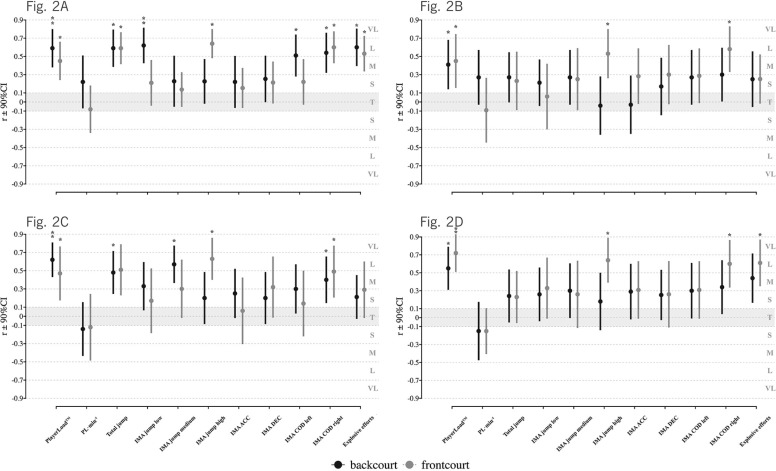
The relationship of RPE and external loads across game quarters based on backcourt and frontcourt players. Note: Fig. 2A: first quarter; Fig. 2B: second quarter, Fig. 2C: third quarter, Fig. 2D: fourth quarter. Effect size categories are denoted as follows: VL = very large; L = large; M = moderate; S = small; and T = trivial; RPE, rating of perceived exertion; PL, PlayerLoad; ACC, acceleration; DEC, deceleration; COD, change of direction; *p ≤ 0.05, **p ≤ 0.01, ***p ≤ 0.001.

Figure 2 illustrates the correlations between internal and external loads across quarters by positions. Across all four quarters, RPE demonstrated consistent and significant positive correlations with PL for backcourt and frontcourt players (backcourt: r = 0.59, 0.41, 0.62, and 0.54; frontcourt: r = 0.46, 0.45, 0.48, and 0.72; all p < 0.05). For frontcourt players, RPE was also significantly correlated with IMA jump high (r = 0.64, 0.53, 0.63, and 0.64; all p < 0.05) and IMA COD right (r = 0.60, 0.58, 0.49, and 0.61; all p < 0.05) across all quarters. Other external metrics, including total jump, IMA jump low/medium, IMA COD left, and explosive efforts, showed moderate-to-large positive associations with RPE in the different quarters, but these relationships were not consistent across all periods, as shown in Figure 2.

**FIG. 3:**
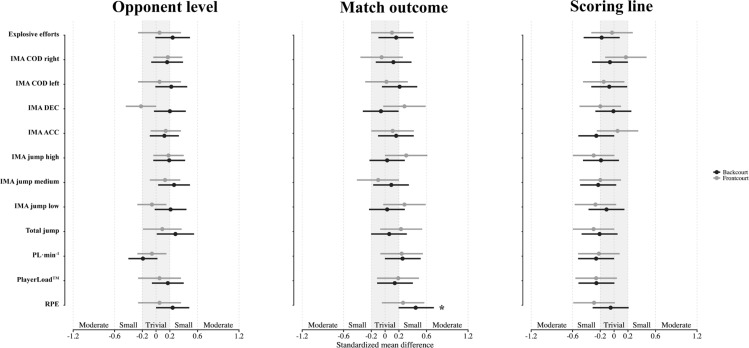
The impact of opponent level, match outcome, and scoring line on backcourt and frontcourt players. Note: RPE, rating of perceived exertion; PL, PlayerLoad; ACC, acceleration; DEC, deceleration; COD, change of direction; *p ≤ 0.05, **p ≤ 0.01, ***p ≤ 0.001

Figure 3 outlines the effects of opponent level, scoring line, and match outcome on external and internal loads by position. Notably, backcourt players reported higher RPE in wins than in losses (ES = 0.42, p < 0.05, small), whereas no significant differences were observed across opponent level or score margin.

## DISCUSSION

The aims of the present study were to 1) investigate the variations in external and internal loads between and within game quarters for backcourt and frontcourt players; 2) explore the relationships between RPE and external loads across quarters in backcourt and frontcourt players, and 3) evaluate the influence of contextual factors on external and internal loads in backcourt and frontcourt players. The key findings indicate that frontcourt players showed significant declines in change-of-direction (IMA COD right) and explosive efforts from the first quarter to the fourth quarter. In contrast, backcourt players recorded highest RPE and PL in the fourth quarter. Within quarters, frontcourt players consistently exhibited higher RPE, PL, and PL · min^−1^, particularly in the second quarter. However, they demonstrated lower values in jump-related metrics (IMA jump low, medium, and total) and IMA ACC compared to backcourt players. Additionally, position-specific analyses revealed distinct load profiles: RPE in backcourt players was significantly associated with PL, whereas RPE in frontcourt players was significantly associated with PL, IMA jump height, and IMA COD right across game quarters. Furthermore, backcourt players reported higher RPE values during winning games compared with losing games. These findings show that loads fluctuate differently across game quarters according to playing position, indicating that in-game monitoring and management should account for position-specific demands.

Significant between-quarter fluctuations were observed in frontcourt players, particularly in the fourth quarter, where a decline in IMA COD right and explosive efforts were observed compared to the first quarter. This finding contrasts with prior research, which reported no significant differences in IMA COD right and explosive efforts across quarters in female collegiate basketball players [41]. Interestingly, one study noted that semi-professional male basketball players performed more IMA COD right movements and total high-intensity events during overtime games than in regulation time [42]. However, our study is the first to demonstrate that professional-level frontcourt players present a decline in IMA COD right and explosive efforts in the fourth quarter than the first quarter, highlighting unique demands in this competitive level. These results underscore the importance of considering position-specific roles, competitive level, and the tactical strategies implemented by coaches when analyzing IMA COD and explosive efforts metrics [43]. Moreover, a decline in IMA COD left and the increase in PL and RPE for backcourt players observed in the later stages of the game may reflect the cumulative effects of fatigue [42]. As the game progresses, maintaining the same activity demands may require greater perceived effort, which is reflected in higher RPE and PL. This pattern may partly reflect a greater contribution from aerobic metabolism to support repeated efforts, along with increased perceptual–cognitive demands imposed by consecutive possessions [44].

Within-quarter comparisons reported significant positional differences in the distribution of external and internal loads across game quarters. Backcourt players presented a lower value in RPE, PL, and PL · min^−1^ in the first half, especially in the second quarter. These findings suggest that the movement profile of backcourt players is characterized by frequent, high-intensity bursts that do not substantially increase overall cumulative load due to their short duration [45].

In contrast, the increased IMA jump performance (e.g. total jump, IMA jump low and medium) observed in backcourt players reflects the frequent execution of vertical movements, which are crucial for transition play and perimeter high-press defense [46]. It is worth noting that backcourt players exhibited more frequent IMA ACC changes from first quarter to four quarter compared to frontcourt players, a finding that is consistent with previous research in U18 female [47] and male professional basketball players [6]. These studies indicate that backcourt players perform more acceleration actions than frontcourt players, likely reflecting their greater involvement in offensive organization, defensive pressure, and repeated one-on-one actions [6]. Our findings extend this part of work, demonstrating that the frequency of IMA ACC actions increases across game quarters, highlighting the higher physical demands placed on backcourt players as the game progresses.

To our knowledge, this study represents the first investigation into the relationship between RPE and external loads based on positional role across game quarters. Our findings demonstrated a moderate to large correlation (r = 0.41–0.72) between RPE and external loads in professional male basketball players. Specifically, RPE exhibited the positive association with PL across all quarters for backcourt players (r = 0.41–0.62) and frontcourt players (r = 0.41–0.72). These results align with prior research [14] utilizing similar measurement equipment, which reported similar correlations (r = 0.49) in semi-professional male basketball players. However, our findings contrast with those observed in professional male basketball players from the Euroleague [48], where a very large correlation (r = 0.82) was noted. Interestingly, a recent study [49] in professional male players from the Chinese National Basketball League reported only small correlations (r = 0.30–0.38) across quarters, which is notably lower than the correlations identified in the present study. Variations in the reported relationships between external and internal loads may stem from differences in player characteristics—such as playing level, experience, and age—which may influence the magnitude of these relationships [50].

Previous studies with same measurement equipment have reported a small to moderate correlation (Euroleague: r = 0.38; NBL: r = 0.25) between sRPE and total jump while a similar relationship (Euroleague: r = 0.45; NBL: r = 0.23) was found between sRPE and IMA jump high [14, 48]. In contrast, our study reported a positive relationship between RPE and total jump across the first two quarters, with correlations ranging from r = 0.48 to 0.59 for backcourt and frontcourt players. Similarly, a significant relationship between RPE and IMA jump high was observed when data were further subdivided by player position. However, it is worth noting that correlations ranging from r = 0.53 to 0.64 were exclusively observed in frontcourt players. These results suggest that positional context should be considered when interpreting the relationship between RPE and jump performance, as it may complement insights reported in previous research [51]. The correlation between RPE and IMA COD to the left and right varied by player position. For backcourt players, correlations ranged from r = 0.40 to 0.60, and for frontcourt players, correlations ranged from r = 0.49 to 0.61, indicating that the magnitude of the relationships was basically similar across positions. To the best of our knowledge, we are the first to demonstrate this relationship between RPE and IMA COD left and right. Although previous studies have reported moderate correlations (r = 0.82) between sRPE and IMA COD metrics, the differences in the selected metrics (e.g. total COD and IMA COD high) and methodologies have prevented direct scientific comparisons or validation of our findings [14, 48]. Consequently, future research should investigate this relationship while accounting for variations in study populations, measurement techniques, and competitive contexts to refine and extend current observation.

Considering contextual factors, backcourt players reported higher RPE in winning games than in losing games. Previous research in both male and female professional basketball has shown that backcourt players typically exhibit higher sRPE and blood lactate levels, primarily due to the high-intensity nature of their responsibilities [12, 45]. This result aligns with a previous study [16] in male semi-professional basketball players, indicating a moderate increase in sRPE in wins compared to losses. This increase may be partly related to psychological demands associated with competitive pressure and decision-making responsibilities during games that are ultimately won [16].

Although this study contributes to understanding the interaction between external and internal loads across game quarters in basketball, several limitations should be considered. First, while the current study provides valuable insights into quarter-to-quarter variations and contextual effects within one season, it cannot fully capture inter-seasonal variability or the influence of different competitive contexts. Future longitudinal studies across multiple seasons are needed to clarify how external and internal loads change over time and interact with different contextual factors, as well as how these patterns relate to performance. Additionally, due to sample size constraints, we were unable to further subdivide the analysis within each quarter to examine how contextual factors influenced the internal and external loads according to specific positions. Another limitation is the reliance on RPE as the primary measure of internal load, as the use of objective markers such as heart rate monitoring or blood lactate sampling was not feasible due to competition regulations. While RPE is widely used due to its practicality and strong correlation with physiological responses [35], it is a subjective measure which may vary between players based on individual perceptions, psychological traits, and contextual factors. To address this, future studies should include objective internal load measures, such as heart rate or blood lactate concentrations, to complement RPE and provide a more comprehensive assessment of internal load. Furthermore, while aligning with approaches adopted in the previous basketball literature [28], we acknowledge that the selected thresholds for high-intensity ACC, DEC, and COD may not be applicable to all basketball teams. Future studies should explore context-specific methods for defining intensity thresholds, such as using percentages of each player’s maximal output [31] or deriving them through cluster-based analyses [52]. Finally, it should be noted that the current study did not examine whether asymmetries in right and left change-of-direction movements are influenced by individual playing style [53]. Future research should consider these factors, as they may provide important insights into individual differences in movement efficiency and performance outcomes.

### Practical application

The present findings offer practical guidance for basketball coaches on managing players more effectively during games. The observed reductions in IMA COD right and explosive efforts in frontcourt players during the fourth quarter may reflect accumulated fatigue or decreased neuromuscular readiness, highlighting the importance of maintaining high-intensity capacity through targeted conditioning. In contrast, backcourt players exhibited progressive increases in RPE and PL across quarters, reflecting the cumulative nature of perceived and mechanical load and highlighting the importance of real-time monitoring to adjust player rotations and avoid excessive fatigue. Moreover, positional differences in the relationships between internal and external loads emphasize the necessity of individualized monitoring strategies; notably, the strong associations between RPE and PL in backcourt and frontcourt players suggest that perceived exertion may serve as a reliable and practical indicator of physical load, enabling coaches to make informed decisions during the competition. Finally, contextual factors such as opponent quality and scoring line appeared to have only limited effects on physical demands, with the exception of slightly higher RPE in backcourt players during wins. This may indicate that positional demands and game progression exert a stronger influence than situational variables. These findings provide a framework for developing position-specific conditioning programs and load management strategies to sustain performance across all quarters.

## CONCLUSIONS

This study examined the dynamics of external and internal loads in professional male basketball players, with particular attention to positional differences between backcourt and frontcourt players across game quarters. Our findings reveal significant fluctuations in load variables, particularly in the fourth quarter, where frontcourt players experienced a significant decline in IMA COD right and explosive efforts, while backcourt players showed the highest RPE and PL. In addition, frontcourt players consistently exhibited higher RPE, PL, and PL · min^−1^, particularly in the second quarter, while demonstrating lower jump-related metrics (IMA jump low, medium, and total) and IMA ACC compared to backcourt players. Our findings also observed position-specific associations between RPE and external load metrics such as PL, total jumps, and COD across game quarters, suggesting that load monitoring and recovery strategies may need to be tailored according to playing position. Notably, backcourt players reported higher RPE values during winning games, further emphasizing the psychophysiological demands associated with positional role. These findings provide actionable insights for coaches and practitioners, suggesting the need for position-specific load management strategies and real-time monitoring to optimize performance and recovery. Future research should examine load dynamics across multiple competitive seasons, incorporate additional objective internal load measures, and expand analyses of contextual factors such as game location and travel demands in order to enhance generalizability and practical applicability.
